# Reward Anticipation Dynamics during Cognitive Control and Episodic Encoding: Implications for Dopamine

**DOI:** 10.3389/fnhum.2016.00555

**Published:** 2016-11-01

**Authors:** Kimberly S. Chiew, Jessica K. Stanek, R. Alison Adcock

**Affiliations:** ^1^Center for Cognitive Neuroscience, Duke UniversityDurham, NC, USA; ^2^Department of Psychology and Neuroscience, Duke UniversityDurham, NC, USA; ^3^Department of Neurobiology, Duke UniversityDurham, NC, USA; ^4^Department of Psychiatry and Behavioral Sciences, Duke University Medical CenterDurham, NC, USA

**Keywords:** dopamine, temporal dynamics, uncertainty, cognitive control, episodic memory

## Abstract

Dopamine (DA) modulatory activity critically supports motivated behavior. This modulation operates at multiple timescales, but the functional roles of these distinct dynamics on cognition are still being characterized. Reward processing has been robustly linked to DA activity; thus, examining behavioral effects of reward anticipation at different timing intervals, corresponding to different putative dopaminergic dynamics, may help in characterizing the functional role of these dynamics. Towards this end, we present two research studies investigating reward motivation effects on cognitive control and episodic memory, converging in their manipulation of rapid vs. multi-second reward anticipation (consistent with timing profiles of phasic vs. ramping DA, respectively) on performance. Under prolonged reward anticipation, both control and memory performances were enhanced, specifically when combined with other experimental factors: task-informative cues (control task) and reward uncertainty (memory task). Given observations of ramping DA under uncertainty (Fiorillo et al., 2003) and arguments that uncertainty may act as a control signal increasing environmental monitoring (Mushtaq et al., 2011), we suggest that task information and reward uncertainty can both serve as “need for control” signals that facilitate learning via enhanced monitoring, and that this activity may be supported by a ramping profile of dopaminergic activity. Observations of rapid (i.e., phasic) reward on control and memory performance can be interpreted in line with prior evidence, but review indicates that contributions of different dopaminergic timescales in these processes are not well-understood. Future experimental work to clarify these dynamics and characterize a cross-domain role for reward motivation and DA in goal-directed behavior is suggested.

Dopamine (DA), a neuromodulator primarily produced in the midbrain and globally broadcast to limbic and cortical targets, is implicated in a range of adaptive behaviors from movement to reward processing to learning. Research characterizing DA function has revealed distinct temporal dynamics—phasic firing and tonic background activity—potentially making separate mechanistic contributions to motivated behavior (Grace, 1991; Niv, 2007). Phasic DA bursts signal unexpected reward, while unexpected withholding of reward depresses phasic DA (positive and negative reward prediction errors; Schultz et al., 1997). These prediction errors may regulate updating of online mental representations (Braver and Cohen, 2000; O’Reilly and Frank, 2006) and guide reward-based learning (Schultz, 1998). Tonic DA is less well-characterized, but, in frontal cortex, may support maintenance of representations (Seamans and Yang, 2004; Westbrook and Braver, 2016), and in striatum, may determine response vigor, or average latency/rate of reward pursuit (Niv et al., 2007).

Recently, a third DA dynamic distinct from both phasic and tonic DA has been identified. Using fast-scan cyclic voltammetry to index rodent striatal DA, Howe et al. (2013) demonstrated that as rodents navigated a spatial maze over a multi-second timescale, moving towards a reward at endpoint, DA ramped with proximity to reward. Further, ramping scaled with reward magnitude. These ramping signals could not be interpreted as phasic activity elicited by prediction error or as tonic background activity, given correspondence to a specific, prolonged epoch of goal pursuit and resolution. This signal has been proposed to be “quasi-tonic” (Lloyd and Dayan, 2015): like tonic DA, ramping DA might operate via extrasynaptic, background firing, but this remains to be comprehensively characterized. In contrast, phasic DA primarily operates via synaptic release (Floresco et al., 2003).

The function of ramping DA remains debated. This ramping dynamic has been observed in rodents traversing a maze towards a reward but also while anticipating a highly-uncertain reward (i.e., 50% reward probability (Fiorillo et al., 2003; Gershman, 2014)) suggested that ramping may reflect averaged activity elicited by reward prediction errors. Evidence from computational models suggests that DA ramps may feasibly support multiple functions: (1) resolution of action timing uncertainty; (2) increasing gain control for a chosen action; and (3) a discounted function of vigor (Lloyd and Dayan, 2015). Importantly, these functions are proposed to be non-mutually exclusive: ramping activity may reflect multiple functions at any given time. Empirical evidence has yet to clarify these accounts.

Given this complexity and present dearth of empirical studies, characterizing the purpose of DA ramping remains a challenge. Functional homology between human and rodent DA systems (Berridge and Kringelbach, 2008) suggests that analogous DA responses can be elicited in humans by similar reward and uncertainty manipulations. Examining human cognitive performance under such manipulations may thus help identify behaviors supported by ramping DA activity and generate more specific experimental hypotheses. Towards that end, we here synthesize findings from two studies showing that manipulating reward anticipation timing (in interaction with other factors: advance task information and reward uncertainty) altered cognitive performance in two distinct cognitive domains: cognitive control and episodic memory. Importantly, reward timing manipulations in both studies contrasted brief (<500 ms) vs. prolonged (multi-second) reward anticipation prior to information processing on each trial; these timing profiles are consistent with time windows previously associated with phasic or ramping DA activity, respectively. We discuss implications of these findings for functional accounts of ramping DA.

Although DA activity and reward anticipation effects have been investigated in multiple cognitive domains, (cognitive control: (Cools, 2008; Locke and Braver, 2008; Krebs et al., 2012; Chiew and Braver, 2013); episodic memory: (Wittmann et al., 2005; Adcock et al., 2006; Daw and Shohamy, 2008; Duzel et al., 2010; Shohamy and Adcock, 2010)), an integrated account of DA in goal-directed behavior requires unifying these observations across functional domains. Traditional models of cognitive control and memory suggest a resource-sharing account whereby increased control is associated with worse subsequent memory (Craik et al., 1996), but more recent investigations indicate that control and memory can work synergistically, with increased top-down control leading to increased memory selectivity for task-relevant information (Richter and Yeung, 2015; Chiu and Egner, 2016). Given that contexts where controlled performance may optimize reward are arguably situations where learning is most critical for future reward-seeking, it is important to investigate for a common role of DA and characterize its potentially synergistic activity across cognitive domains.

## Study 1: Reward Timing, Task Information, and Cognitive Control (Chiew and Braver, 2016)

**Figure 1:**
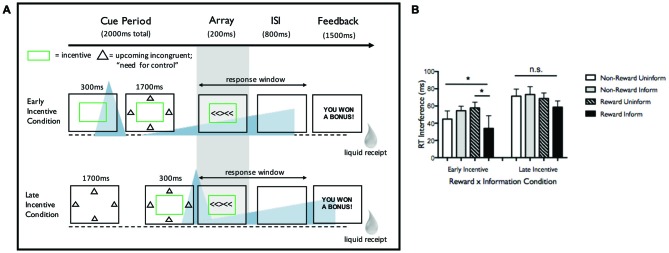
**(A)** Task design from Experiment 2 from Chiew and Braver (2016). Incentive and task-informative cues were manipulated on a trial-by-trial basis, while the two timing conditions (Early and Late Incentive) were blocked. This figure shows an incentivized, task-informed trial for both Early Incentive and Late Incentive conditions. In the Early Incentive condition, participants were first presented with a rectangle (with rectangle color indicating incentive status—green for incentive trials, white for non-incentive trials), followed by informative (shapes surrounding the rectangle indicating upcoming congruent, neutral, or incongruent array) or uninformative (question marks surrounding the rectangle) cue, followed by target (flanker array). In the Late Incentive condition, participants were presented with the informative/uninformative cue first, followed by incentive/unincentive cue, followed by target. Importantly, informative cues indicated upcoming trial status but not the direction of the flanker arrow, so participants could not use the information to prepare a motor response. Participants were explicitly instructed on the meaning of the incentive and task-informative cues and tested both before and after the task to ensure that cues had been learned. Participants were required to respond prior to an individualized reaction time criterion (30th percentile of correct reaction times from a prior baseline; 1000 ms total response window), then received liquid feedback and a 2000 ms ITI. Participants were rewarded only when accurate and faster than criterion. The average reward rate was 75% (range: 46–96%) under Early Incentive and 71% (range: 41–99%) under Late Incentive, compared to an expected reward rate of 30% at baseline performance, indicating that the incentives enhanced overall performance. **(B)** Reaction time (RT) interference in Experiment 2 as a function of incentive and task information. Asterisks indicate significant effects (*p* < 0.05). In the Early Incentive condition, a significant Incentive × Information interaction was observed such that interference was lowest in incentivized, informed trials. Main effects of Incentive and Information were not significant. In the Late Incentive condition, no significant differences in interference as a function of these factors were observed. While Incentive and Information led to different effects within timing condition as noted, it should be noted that a full three-way interaction of Timing × Incentive × Information was not significant (*F*_(1,23)_ = 1.864, *p* = 0.185).

This study examined Eriksen flanker task performance as a function of reward and task-informative (deterministically predicting congruent, neutral, or incongruent array) cues in two experiments. We focus on Experiment 2 here (paradigm and key findings in Figure 1). Both experiments demonstrated that task-informative cues, specifically when combined with reward incentive (awarded for fast, accurate responses), led to reduced interference (incongruent minus neutral reaction times; lower interference indicates enhanced control). In Experiment 2, manipulating cue timing prior to target modulated these effects. When reward cue was presented 2000 ms before flanker array (“Early Incentive” condition), enhanced cognitive control was observed, with a specific benefit for informed trials. In contrast, when reward cue was presented 300 ms before flanker array (“Late Incentive” condition), participants sped up under incentive^1^ but interference costs did not significantly change with incentive or task information. These results were interpreted as evidence that participants can use preparatory information to up-regulate control, but only when motivated to do so (i.e., by incentive), and only with adequate time to engage cue-constrained attention.

Consideration of dopaminergic dynamics elicited by reward cue at these different timing intervals may help elucidate the mechanisms underlying behavioral outcome. In the Late Incentive condition (reward cue 300 ms prior to target), flanker performance would occur during the putative time-window for phasic DA activity, while in the Early Incentive condition (with reward cue 2000 ms prior to target) flanker performance would occur during the putative time-window for DA ramping (following the anticipation interval in Fiorillo et al., 2003). In the memory domain, Stanek et al. (submitted) used a similar reward timing manipulation, evoking putative phasic vs. ramping DA responses, and examined how timing interacted with reward probability to influence encoding. We discuss this study and its findings below, then consider both studies and their collective implications for the effects of reward anticipation dynamics on cognition.

## Study 2: Reward Timing, Probability and Episodic Memory (Stanek et al., submitted)

**Figure 2:**
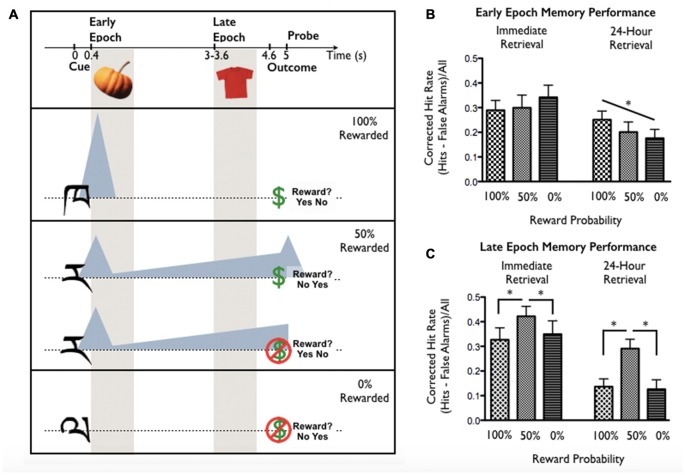
**(A)** Task design from Stanek et al. (submitted). The task was designed to dissociate two putative Dopamine (DA) dynamics during reward anticipation—a rapid phasic response scaling with expected reward value, and a prolonged response that increases with uncertainty (following Fiorillo et al., 2003). These profiles are indicated by shaded triangles relative to trial events. Reward certainty and stimulus presentation epoch were manipulated trial-to-trial. Cues associated with 100%, 50%, or 0% reward probability were presented for 400 ms. Cue-reward relationships were learned and tested prior to task performance. Incidental encoding objects were presented either immediately following the cue (Early Epoch; 400 ms anticipation period) or shortly before the anticipated reward outcome (Late Epoch; 3000–3600 ms anticipation period). Following reward outcome, participants responded to a probe (yes/no answer; location counterbalanced) asking whether or not they had received a reward. **(B)** Early Epoch memory performance linearly increased with expected reward value in the 24-h retrieval group (performance 100% > 50% > 0%) but not in the immediate encoding group. **(C)** Late Epoch memory performance was greatest for items encoded during reward uncertainty (50% > 100% and 50% > 0%) in both the immediate and 24-h retrieval groups. Asterisks indicate significant effects (*p < 0.05*).

This study examined incidental memory for stimuli shown during reward anticipation as a function of reward probability, timing relative to reward cue onset, and retention interval. On each trial, participants were cued to anticipate a reward by (previously-learned) abstract cues signaling 0%, 50%, or 100% reward probability. Participants viewed incidental object stimuli either early in the anticipation epoch (Early Epoch; immediately after cue offset, 400 ms post-cue onset) or late in the anticipation epoch (Late Epoch; immediately before reward outcome, 3000–3600 ms post-cue onset). The critical anticipation period was between reward cue and incidental stimulus: Early and Late Epoch intervals corresponded to time windows associated with phasic DA, anticipated to scale with expected reward value, or prolonged ramping DA, anticipated to scale with reward uncertainty (following Fiorillo et al., 2003). Participant object memory was tested 15 min or 24 post-encoding (assessing memory without and with consolidation). The paradigm and putative DA dynamics are shown in Figure 2A.

For objects presented at Early Epoch (Figure 2B), memory benefit scaled with reward probability (100% > 50% > 0%) but only post-consolidation (24-h retention). In contrast, for objects presented at Late Epoch (Figure 2C), memory benefit was found under reward uncertainty (50% > 100% and 50% > 0%) at both retention intervals. These findings suggest that expected reward value and uncertainty may have dissociable influences on memory formation: timing of the memory benefit for expected reward value was consistent with putative phasic DA (occurring at Early but not Late Epoch) and was consolidation-dependent, while memory benefit for uncertainty was consistent with putative ramping DA (occurring at Late but not Early Epoch) and was consolidation-independent.

## Reward Anticipation Dynamics: Synthesizing Across Findings

While these studies were conducted with different original aims (examining reward interactions with probability vs. advance task information), in distinct cognitive domains, manipulations of reward timing across the two studies provide parallel contrasts of putative dopaminergic dynamics. Reward anticipation prior to target in Chiew and Braver’s Early Incentive condition (Experiment 2) and object encoding in Stanek et al.’s Late Epoch both correspond to the timescale associated with prolonged, ramp-like DA responses during reward anticipation: 2–8 s (Fiorillo et al., 2003; Howe et al., 2013; Totah et al., 2013). In contrast, reward anticipation prior to target in Chiew and Braver’s Late Incentive condition (Experiment 2) and object encoding in Stanek et al.’s (submitted) Early Epoch both correspond to the timescale for phasic DA following reward cue (<500 ms; Schultz et al., 1997). Given similarities in reward timing, considering the effects of these studies together may help inform the functions of prolonged and phasic DA across cognitive domains.

### Cognitive Performance During Putative DA Ramping

Prolonged reward anticipation was associated both with enhanced control (Chiew and Braver, 2016) and with enhanced memory for incidental stimuli (Stanek et al., submitted); however, in both studies, these effects were specifically seen in interaction with other experimental factors. Chiew and Braver demonstrated that while interference was lower under prolonged vs. brief reward anticipation (Early vs. Late Incentive), a specific control benefit was observed in Early Incentive informed trials. Stanek et al. (submitted) demonstrated enhanced memory for objects presented after prolonged reward anticipation (at Late Epoch) specifically under high reward uncertainty (50%, vs. 0% or 100%, probability).

These experimental manipulations are obviously different—task-informative cues arguably reduced task uncertainty in the control task, while the 50% cue increased reward uncertainty in the memory task. However, we argue that both the task-informative cue and the reward uncertainty cue may act as “need-for-control” signals facilitating learning via increased environmental monitoring, yielding convergent benefits to both control and memory, specifically under prolonged reward anticipation. A recent review argues that uncertainty may overlap with cognitive control conceptually and neurally (Mushtaq et al., 2011): when upcoming events are highly unpredictable, this uncertainty indicates the need to actively regulate and update environmental representations to improve predictions and optimize behavior. Consistent with this, increasing perceptual uncertainty has been associated with increased monitoring-related activity in ventral striatum and with shifts in task strategy (Buzzell et al., 2016).

Thus, both uncertainty and task-informative cues may serve as examples of signals indicating the need and opportunity for increased control. We further suggest that these elicitors of control indicate that upcoming stimuli are goal-relevant and prime the cognitive system for enhanced learning, given evidence that increased control enhances subsequent memory for task-relevant stimuli (Richter and Yeung, 2015). These observations lead to testable hypotheses that both uncertainty and explicit control signals under prolonged reward anticipation will benefit cognitive control and subsequent memory for task-relevant information, and that these benefits should be supported by prolonged, ramping dopaminergic activity.

### Cognitive Performance During Putative Phasic DA

When a reward cue was presented <500 ms before target in Chiew and Braver (2016), response speeding was observed in incentive vs. non-incentive trials but interference did not decrease.This suggests that incentives did not enhance control under these conditions. When reward cue was presented <500 ms before incidental stimuli in Stanek et al. (submitted), subsequent memory for those stimuli scaled with expected reward value (100% > 50% > 0% reward probability) but only post-consolidation (24-interval).

Phasic DA has been proposed as a mechanism by which representations are updated in working memory as well as a means by which value functions update in reinforcement learning (Westbrook and Braver, 2016). Our results under rapid reward cueing—response speeding without increased control, and enhanced episodic memory performance post-consolidation—do not correspond neatly to these purported functions. Some evidence suggests that DA modulates response speeding—decision thresholds during speeded response may be mediated by a DA-modulated cortico-basal ganglia network (Lo and Wang, 2006), and reward-related improvement in both accuracy and speed, “breaking” the speed-accuracy tradeoff, may be attributable to DA-based effort deployment (Manohar et al., 2015). However, Chiew and Braver (2016) used a strict time cutoff which may have prohibited reward-based “breaking the tradeoff”; additional evidence suggests that DA manipulations do not always alter speed-accuracy tradeoff (Winkel et al., 2012). Further, these investigations did not disentangle possible DA roles at different temporal dynamics. Thus, while this literature indicates a potential role for DA in adjusting response speeding, considerable ambiguity remains.

Regarding episodic memory, it has been argued that DA input to hippocampus operates on multiple timescales (Lisman and Grace, 2005; Shohamy and Adcock, 2010), but potentially separate influences of phasic and sustained DA effects on episodic memory have yet to be comprehensively characterized. Stanek et al. present evidence for reward effects on episodic memory, corresponding to phasic DA, that scale with expected reward value and depend on consolidation. This finding is consistent with observations of a relationship between DA and consolidation-dependent effects on memory (Bethus et al., 2010; McNamara et al., 2014; Gruber et al., 2016) but expands on these findings with novel evidence that the DA-memory relationship might differ as a function of temporal dynamics. Together, our control and memory results under phasic reward can be considered consistent with prior research, but these findings are also somewhat disparate and difficult to integrate with each other. We suggest that this reflects important gaps in existing literature. Specifically, phasic DA effects on learning have been discussed largely in terms of reward prediction errors, and timing-specific DA effects on control or episodic encoding have not been clearly delineated. Further investigation is needed to characterize these potentially distinct effects and facilitate conceptual integration across cognitive domains.

## Future Directions

We review these two empirical studies side-by-side to compare effects of prolonged vs. brief reward anticipation, eliciting putative ramping and phasic DA respectively, on cognitive control and episodic memory encoding. We argue that this comparison reveals important potential commonalities in reward anticipation effects between the two studies, as well as highlighting directions for future research. We summarize these points below.

Both memory and control performance was enhanced for stimuli following prolonged reward anticipation, specifically when combined with reward uncertainty (memory task) and advance information of control demand (control task). On this timescale, DA neurons have been shown to exhibit gradually increasing (ramping) anticipatory activity, scaling with reward uncertainty (Fiorillo et al., 2003). Given prior argument that uncertainty serves to signal cognitive control demand (Mushtaq et al., 2011), we suggest that a similar ramping signal could have supported enhanced control, in response to task-informative cues, in Chiew and Braver (2016). Potential commonalities in cognitive control and memory effects following brief reward anticipation (corresponding to phasic DA) are less straightforward, but observed control performance (response speeding without enhanced control) and memory performance (enhanced consolidation-dependent memory scaling with expected reward value) dovetail with prior evidence for DA involvement in response speeding and episodic encoding. In all, however, prior literature suggests that distinct functional roles of different DA dynamics (phasic vs. ramping vs. tonic) in supporting cognition have not been clearly delineated. Future work will be needed to address this conceptual gap. We offer more specific predictions for cross-domain investigations clarifying a potential role for prolonged, ramping DA across control and memory processes below.

First, our argument that both uncertainty and task-informative cues might elicit cognitive control and prime the cognitive system for enhanced learning leads to the hypothesis that, in a cognitive control task such as the one used by Chiew and Braver (2016), memory should be enhanced for target stimuli under conditions where high control is elicited. Specifically, under the combination of prolonged reward anticipation and task-informative cue, task control and subsequent memory for target stimuli may be highest, and supported by a ramping dopaminergic response. Further, given results that increased control can increase memory selectivity for task-relevant vs. irrelevant stimuli (Richter and Yeung, 2012; Chiu and Egner, 2016), we hypothesize that memory benefit will be specific to task-relevant stimuli. Using a control paradigm with targets and distractors, such as our flanker task (Chiew and Braver, 2016), with more complex, encode-able stimuli and a subsequent memory test would allow testing of this hypothesis. Likewise, it would be interesting to examine whether reward uncertainty during a prolonged anticipation period would lead to enhanced control. These investigations could help clarify whether uncertainty should be considered a “need-for-control” signal and its behavioral influence across multiple cognitive domains.

These investigations further set up predictions regarding DA dynamics during reward anticipation. While ramping DA signals have not yet been demonstrated in humans, our laboratory recently demonstrated distinct timescales of activity in human ventral tegmental area (VTA), the primary source of forebrain DA, during novelty processing (Murty et al., 2016). Additionally, ramping activity dynamics have been explicitly modeled in rostrolateral prefrontal cortex as a function of task sequence position (Desrochers et al., 2015). Characterizing a human ramping DA response could potentially build on these approaches.

## Conclusion

In this perspective article, we reviewed two studies, examining cognitive control and episodic memory performance, using parallel reward anticipation manipulations potentially capturing prolonged vs. phasic dopaminergic responses. Examining behavior as a function of reward anticipation timing across these two paradigms may help elucidate influences of prolonged vs. phasic DA on cognition and what experimental factors may be important to eliciting these states. We argue that the role of phasic DA in functions beyond reinforcement learning needs to be more clearly characterized and suggest future experimental work to further investigate potential effects of ramping DA on behavior. This work will help improve characterization of dopaminergic effects at different timescales and their influences on cognition, advancing our understanding of goal-directed behavior.

## Author Contributions

KSC, JKS and RAA conceived and wrote the article.

## Funding

This study was supported by a postdoctoral fellowship from the Canadian Institutes of Health Research to KSC (MFE-135441).

## Conflict of Interest Statement

The authors declare that the research was conducted in the absence of any commercial or financial relationships that could be construed as a potential conflict of interest.

## Footnotes

^1^In the Late Incentive condition, response speeding under trial-by-trial incentive occurred in all trial types (incongruent, neutral and congruent) without specifically benefiting incongruent trials (which would have led to a decrease in interference costs). Refer to Chiew and Braver (2016) for statistics.

## References

[B1] Adcock R. A. Thangavel A. Whitfield-Gabrieli S. Knutson B. Gabrieli J. D. (2006). Reward-motivated learning: mesolimbic activation precedes memory formation. Neuron 50, 507–517. 10.1016/j.neuron.2006.03.036 16675403

[B2] Berridge K. C. Kringelbach M. L. (2008). Affective neuroscience of pleasure: reward in humans and animals. Psychopharmacology (Berl) 199, 457–480. 10.1007/s00213-008-1099-6 18311558 PMC3004012

[B3] Bethus I. Tse D. Morris R. G. (2010). Dopamine and memory: modulation of the persistence of memory for novel hippocampal NMDA receptor-dependent paired associates. J. Neurosci. 30, 1610–1618. 10.1523/JNEUROSCI.2721-09.2010 20130171 PMC6633999

[B4] Braver T. S. Cohen J. D. (2000). “On the control of control: the role of dopamine in regulating prefrontal function and working memory,” in Attention and Performance XVIII, eds Monsell S. Driver J. (Cambridge, MA: MIT Press), 713–737.

[B5] Buzzell G. A. Roberts D. M. Fedota J. R. Thompson J. C. Parasuraman R. McDonald C. G. (2016). Uncertainty-dependent activity within the ventral striatum predicts task-related changes in response strategy. Cogn. Affect. Behav. Neurosci. 16, 219–233. 10.3758/s13415-015-0383-2 26453582

[B6] Chiew K. S. Braver T. S. (2013). Temporal dynamics of motivation-cognitive control interactions revealed by high-resolution pupillometry. Front. Psychol. 4:15. 10.3389/fpsyg.2013.00015 23372557 PMC3557699

[B7] Chiew K. S. Braver T. S. (2016). Reward favors the prepared: incentive and task-informative cues interact to enhance attentional control. J. Exp. Psychol. Hum. Percept. Perform. 42, 52–66. 10.1037/xhp0000129 26322689 PMC4688088

[B8] Chiu Y.-C. Egner T. (2016). Distractor-relevance determines whether task-switching enhances or impairs distractor memory. J. Exp. Psychol. Hum. Percept. Perform. 42, 1–5. 10.1037/xhp0000181 26594883 PMC4688095

[B9] Cools R. (2008). Role of dopamine in the motivational and cognitive control of behavior. Neuroscientist 14, 381–395. 10.1177/1073858408317009 18660464

[B10] Craik F. I. Govoni R. Naveh-Benjamin M. Anderson N. D. (1996). The effects of divided attention on encoding and retrieval processes in human memory. J. Exp. Psychol. Gen. 125, 159–180. 10.1037/0096-3445.125.2.159 8683192

[B11] Daw N. D. Shohamy D. (2008). The cognitive neuroscience of motivation and learning. Soc. Cogn. 26, 593–620. 10.1521/soco.2008.26.5.593

[B12] Desrochers T. M. Chatham C. H. Badre D. (2015). The necessity of rostrolateral prefrontal cortex for higher-level sequential behavior. Neuron 87, 1357–1368. 10.1016/j.neuron.2015.08.026 26402612 PMC4630805

[B13] Duzel E. Bunzeck N. Guitart-Masip M. Düzel S. (2010). NOvelty-related motivation of anticipation and exploration by dopamine (NOMAD): implications for healthy aging. Neurosci. Biobehav. Rev. 34, 660–669. 10.1016/j.neubiorev.2009.08.006 19715723

[B14] Fiorillo C. D. Tobler P. N. Schultz W. (2003). Discrete coding of reward probability and uncertainty by dopamine neurons. Science 299, 1898–1902. 10.1126/science.1077349 12649484

[B15] Floresco S. B. West A. R. Ash B. Moore H. Grace A. A. (2003). Afferent modulation of dopamine neuron firing differentially regulates tonic and phasic dopamine transmission. Nat. Neurosci. 6, 968–973. 10.1038/nn1103 12897785

[B16] Gershman S. J. (2014). Dopamine ramps are a consequence of reward prediction errors. Neural Comput. 26, 467–471. 10.1162/NECO_A_00559 24320851

[B17] Grace A. A. (1991). Phasic versus tonic dopamine release and the modulation of dopamine system responsivity: a hypothesis for the etiology of schizophrenia. Neuroscience 41, 1–24. 10.1016/0306-4522(91)90196-u 1676137

[B18] Gruber M. J. Ritchey M. Wang S.-F. Doss M. K. Ranganath C. (2016). Post-learning hippocampal dynamics promote preferential retention of rewarding events. Neuron 89, 1110–1120. 10.1016/j.neuron.2016.01.017 26875624 PMC4777629

[B19] Howe M. W. Tierney P. L. Sandberg S. G. Phillips P. E. Graybiel A. M. (2013). Prolonged dopamine signalling in striatum signals proximity and value of distant rewards. Nature 500, 575–579. 10.1038/nature12475 23913271 PMC3927840

[B21] Krebs R. M. Boehler C. N. Roberts K. C. Song A. W. Woldorff M. G. (2012). The involvement of the dopaminergic midbrain and cortico-striatal-thalamic circuits in the integration of reward prospect and attentional task demands. Cereb. Cortex 22, 607–615. 10.1093/cercor/bhr134 21680848 PMC3278318

[B22] Lisman J. E. Grace A. A. (2005). The hippocampal-VTA loop: controlling the entry of information into long-term memory. Neuron 46, 703–713. 10.1016/j.neuron.2005.05.002 15924857

[B23] Lloyd K. Dayan P. (2015). Tamping ramping: algorithmic, implementational and computational explanations of phasic dopamine signals in the accumbens. PLoS Comput. Biol. 11:e1004622. 10.1371/journal.pcbi.1004622 26699940 PMC4689534

[B24] Lo C. C. Wang X. J. (2006). Cortico-basal ganglia circuit mechanism for a decision threshold in reaction time tasks. Nat. Neurosci. 9, 956–963. 10.1038/nn1722 16767089

[B25] Locke H. S. Braver T. S. (2008). Motivational influences on cognitive control: behavior, brain activation and individual differences. Cogn. Affect. Behav. Neurosci. 8, 99–112. 10.3758/cabn.8.1.99 18405050

[B26] Manohar S. G. Chong T. T. Apps M. A. Batla A. Stamelou M. Jarman P. R.. (2015). Reward pays the cost of noise reduction in motor and cognitive control. Curr. Biol. 25, 1707–1716. 10.1016/j.cub.2015.05.038 26096975 PMC4557747

[B27] McNamara C. G. Tejero-Cantero A. Trouche S. Campo-Urriza N. Dupret D. (2014). Dopaminergic neurons promote hippocampal reactivation and spatial memory persistence. Nat. Neurosci. 17, 1658–1660. 10.1038/nn.3843 25326690 PMC4241115

[B28] Murty V. P. Ballard I. C. Adcock R. A. (2016). Hippocampus and prefrontal cortex predict distinct timescales of activation in the human ventral tegmental area. Cereb. Cortex [Epub ahead of print]. 10.1093/cercor/bhw005 26826101 PMC6075214

[B29] Mushtaq F. Bland A. R. Schaefer A. (2011). Uncertainty and cognitive control. Front. Psychol. 2:249. 10.3389/fpsyg.2011.00249 22007181 PMC3184613

[B30] Niv Y. (2007). Cost, benefit, tonic, phasic: what do response rates tell us about dopamine and motivation? Ann. N Y Acad. Sci. 1104, 357–376. 10.1196/annals.1390.018 17416928

[B31] Niv Y. Daw N. D. Joel D. Dayan P. (2007). Tonic dopamine: opportunity costs and the control of response vigor. Psychopharmacology (Berl) 191, 507–520. 10.1007/s00213-006-0502-4 17031711

[B32] O’Reilly R. C. Frank M. J. (2006). Making working memory work: a computational model of learning in the prefrontal cortex and basal ganglia. Neural Comput. 18, 283–328. 10.1162/089976606775093909 16378516

[B33] Richter F. R. Yeung N. (2012). Memory and cognitive control in task switching. Psychol. Sci. 23, 1256–1263. 10.1177/0956797612444613 22972906

[B34] Richter F. R. Yeung N. (2015). Corresponding influences of top-down control on task switching and long-term memory. Q. J. Exp. Psychol. (Hove) 68, 1124–1147. 10.1080/17470218.2014.976579 25337969

[B35] Schultz W. (1998). Predictive reward signal of dopamine neurons. J. Neurophysiol. 80, 1–27. 9658025 10.1152/jn.1998.80.1.1

[B36] Schultz W. Dayan P. Montague P. R. (1997). A neural substrate of prediction and reward. Science 275, 1593–1599. 10.1126/science.275.5306.1593 9054347

[B37] Seamans J. K. Yang C. R. (2004). The principal features and mechanisms of dopamine modulation in the prefrontal cortex. Prog. Neurobiol. 74, 1–58. 10.1016/j.pneurobio.2004.05.006 15381316

[B38] Shohamy D. Adcock R. A. (2010). Dopamine and adaptive memory. Trends Cogn. Sci. 14, 464–472. 10.1016/j.tics.2010.08.002 20829095

[B40] Totah N. K. Kim Y. Moghaddam B. (2013). Distinct prestimulus and poststimulus activation of VTA neurons correlates with stimulus detection. J. Neurophysiol. 110, 75–85. 10.1152/jn.00784.2012 23554430 PMC3727034

[B41] Westbrook A. Braver T. S. (2016). Dopamine does double duty in motivating cognitive effort. Neuron 89, 695–710. 10.1016/j.neuron.2015.12.029 26889810 PMC4759499

[B42] Winkel J. van Maanen L. Ratcliff R. van der Schaaf M. E. van Schouwenburg M. R. Cools R.. (2012). Bromocriptine does not alter speed-accuracy tradeoff. Front. Neurosci. 6:126. 10.3389/fnins.2012.00126 22969702 PMC3430867

[B43] Wittmann B. C. Schott B. H. Guderian S. Frey J. U. Heinze H. J. Düzel E. (2005). Reward-related FMRI activation of dopaminergic midbrain is associated with enhanced hippocampus-dependent long-term memory formation. Neuron 45, 459–467. 10.1016/j.neuron.2005.01.010 15694331

